# Hypersensitivity of myelinated A-fibers via toll-like receptor 5 promotes mechanical allodynia in tenascin-X-deficient mice associated with Ehlers–Danlos syndrome

**DOI:** 10.1038/s41598-023-45638-7

**Published:** 2023-10-28

**Authors:** Hiroki Kamada, Kousuke Emura, Rikuto Yamamoto, Koichi Kawahara, Sadahito Uto, Toshiaki Minami, Seiji Ito, Ken-ichi Matsumoto, Emiko Okuda-Ashitaka

**Affiliations:** 1 Department of Biomedical Engineering, Osaka Institute of Technology, Osaka, 535-8585 Japan; 2 Department of Anesthesiology, Osaka Medical and Pharmaceutical University, Takatsuki, 569-8686 Japan; 3 Department of Biosignaling and Radioisotope Experiment, Interdisciplinary Center for Science Research, Head Office for Research and Academic Information, Shimane University, Izumo, 693-8501 Japan

**Keywords:** Neuroscience, Molecular medicine, Neurology

## Abstract

Deficiency of an extracellular matrix glycoprotein tenascin-X (TNX) leads to a human heritable disorder Ehlers–Danlos syndrome, and TNX-deficient patients complain of chronic joint pain, myalgia, paresthesia, and axonal polyneuropathy. We previously reported that TNX-deficient (*Tnxb*^−/*−*^) mice exhibit mechanical allodynia and hypersensitivity to myelinated A-fibers. Here, we investigated the pain response of *Tnxb*^−/*−*^ mice using pharmacological silencing of A-fibers with co-injection of *N*-(2,6-Dimethylphenylcarbamoylmethyl) triethylammonium bromide (QX-314), a membrane-impermeable lidocaine analog, plus flagellin, a toll-like receptor 5 (TLR5) ligand. Intraplantar co-injection of QX-314 and flagellin significantly increased the paw withdrawal threshold to transcutaneous sine wave stimuli at frequencies of 250 Hz (Aδ fiber responses) and 2000 Hz (Aβ fiber responses), but not 5 Hz (C fiber responses) in wild-type mice. The QX-314 plus flagellin-induced silencing of Aδ- and Aβ-fibers was also observed in *Tnxb*^−/*−*^ mice. Co-injection of QX-314 and flagellin significantly inhibited the mechanical allodynia and neuronal activation of the spinal dorsal horn in *Tnxb*^−/*−*^ mice. Interestingly, QX-314 alone inhibited the mechanical allodynia in *Tnxb*^−/*−*^ mice, and it increased the paw withdrawal threshold to stimuli at frequencies of 250 Hz and 2000 Hz in *Tnxb*^−/*−*^ mice, but not in wild-type mice. The inhibition of mechanical allodynia induced by QX-314 alone was blocked by intraplantar injection of a TLR5 antagonist TH1020 in *Tnxb*^−/*−*^ mice. These results suggest that mechanical allodynia due to TNX deficiency is caused by the hypersensitivity of Aδ- and Aβ-fibers, and it is induced by constitutive activation of TLR5.

## Introduction

Tenascin-X (TNX) is an extracellular matrix glycoprotein, and TNX deficiency caused by compound heterozygous and homozygous mutations of the gene leads to a heritable connective tissues disorder Ehlers–Danlos syndrome (EDS)^1–5^. EDS is characterized by hyperextensibility of skin, hypermobility of joints, and fragility of various connective tissues. In addition, pain is a sever clinical manifestation of EDS patients, and approximately 90% of EDS patients complain of chronic pain^6–8^. EDS patients suffer from chronic neuropathic pain, generalized body pain, gastrointestinal pain, headache, dysmenorrhea, and fatigue, in addition to connective tissues-related pain such as joint pain, soft-tissue pain, and dislocations^6, 7^. TNX-deficient EDS patients complain of chronic pain such as chronic joint pain, myalgia, abdominal pain, fatigue, paresthesia, and axonal polyneuropathy^3, 5^. We have previously reported the pain behaviors of TNX-deficient (*Tnxb*^−/*−*^) mice as a murine EDS model^9^. *Tnxb*^−/*−*^ mice exhibited increased sensitivity to innocuous mechanical stimuli, which is called mechanical allodynia, a major feature of neuropathic pain. *Tnxb*^−/*−*^ mice also showed significant hypersensitivity to transcutaneous sine wave stimuli at frequencies of 250 Hz (myelinated Aδ fiber responses) and 2000 Hz (myelinated Aβ fiber responses) using analysis of sensory afferent fiber responses to a sine wave electric stimulator. However, the hypersensitivity of myelinated Aδ- and Aβ-fibers by which TNX deficiency complicates the mechanical allodynia remains unknown.

It has been reported that blocking A-fibers can be specifically achieved by using the toll-like receptor 5 (TLR5) ligand flagellin and the membrane-impermeable sodium channel blocker *N*-(2,6-Dimethylphenylcarbamoylmethyl) triethylammonium bromide (QX-314)^10^. Activation of TLR5 induced by flagellin results in neuronal entry of QX-314, provoking TLR5-dependent blockade of sodium currents, primarily in Aβ fibers. Intraplantar co-administration of flagellin and QX-314 suppressed mechanical allodynia in neuropathic pain models induced by chemotherapy, nerve injury, and diabetic neuropathy. On the other hand, blocking C-fibers can be specifically achieved using the transient receptor potential vanilloid 1 (TRPV1) ligand capsaicin and QX-314^11^. Co-administration of capsaicin and QX-314 resulted in the neuronal entry of QX-314 through the ion channel TRPV1, provoking a blockade of C-fibers.

In the present study, we used fiber-specific blockade using co-injection of QX-314 with flagellin or capsaicin to *Tnxb*^−/*−*^ mice, in order to elucidate the correlation between TNX deficiency-induced hypersensitivity of myelinated A-fibers and the mechanical allodynia. We examined the combination of sensory afferent fiber responses to a sine wave electric stimulator and behavioral pharmacology in *Tnxb*^−/*−*^ mice injected with QX-314, flagellin, or capsaicin. We demonstrated that TNX-deficiency-induced mechanical allodynia is mediated by A-fiber hypersensitivity, and that QX-314 alone-inhibited mechanical allodynia is mediated by constitutive activation of TLR5 in *Tnxb*^−/*−*^ mice.

## Results

### Sensory fiber-specific silencing in vivo in wild-type mice

**Figure 1:**
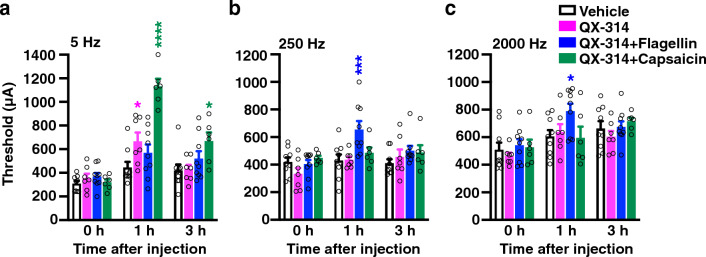
Effect of pharmacological sensory fiber-specific silencing on the responses of sensory afferent fibers using transcutaneous sine wave stimuli in *Tnxb*^+*/*+^mice. The current threshold represents the minimum intensity (μA) required to produce a paw withdrawal response with sine wave electrical stimulation at 5 Hz (**a**), 250 Hz (**b**), and 2000 Hz (**c**) in mice at the indicated time after intraplantar (i.pl.) administration of vehicle, QX-314 (30 mM), QX-314 (30 mM) plus flagellin (0.5 μg) for A-fiber silencing, and QX-314 (30 mM) plus capsaicin (10 μg) for C-fiber silencing. Vehicle (n = 9), QX-314 (n = 7), QX-314 + flagellin (n = 9), and QX-314 + capsaicin (n = 6). ^****^P < 0.0001, ^***^P < 0.001, ^*^P < 0.05 versus the vehicle-treated value, two-way repeated measurement ANOVA revealed a significant treatment (5 Hz: F_(3,27)_ = 18.44, *P* < 0.0001; 250 Hz: F_(3,27)_ = 3.266, *P* = 0.0366; 2000 Hz: F_(3,27)_ = 3.118, *P* = 0.0425) with post hoc Bonferroni’s multiple comparison test. The data are expressed as the mean ± S.E.M.

We used pharmacological A-fiber silencing by intraplantar co-injection of the membrane-impermeable sodium channel blocker QX-314 and the TLR5 ligand flagellin, and C-fiber silencing by QX-314 and the TRPV1 ligand capsaicin. Intraplantar application of QX-314 (2%, approximately 60 mM, 20 μL) with flagellin (0.3 μg in 20 μL) suppressed A-fiber conduction in naive and chemotherapy-treated mice, and application of QX-314 (0.2%, approximately 6 mM) with flagellin (0.1–0.9 μg) inhibited mechanical allodynia in neuropathic pain models induced by chemotherapy^10^. Intraplantar injection of QX-314 (2%, approximately 60 mM; 10 μL) and capsaicin (1 μg/μL; 10 μL) together produced long-lasting increase in mechanical and thermal nociceptive thresholds^11^. We first examined whether sensory fiber-specific silencing in vivo affected the responses of sensory afferent fibers by using transcutaneous sine wave stimuli in wild-type mice. Transcutaneous nerve stimulation was conducted using three sine wave pulses with frequencies of 5, 250, and 2000 Hz to activate the C, Aδ, and Aβ fibers, respectively^12, 13^. As shown in Fig. 1, when 20 μL of QX-314 (30 mM) with flagellin (0.5 μg in 20 μL) were simultaneously injected into the subcutaneous plantar hind paw of wild-type mice, the paw withdrawal threshold to stimuli at frequencies of 250 Hz and 2000 Hz, but not 5 Hz, were significantly increased at 1 h after the injection compared to the vehicle-treated mice (Fig. 1b, 250 Hz: vehicle: 430 ± 44 μA (n = 9) vs. QX-314 + flagellin: 654 ± 61 μA (n = 9), *P* = 0.0005; Fig. 1c, 2000 Hz: vehicle: 604 ± 47 μA vs. QX-314 + flagellin: 789 ± 55 μA, *P* = 0.0406). Intraplantar co-injection of QX-314 (30 mM) and capsaicin (10 μg in 20 μL) increased the paw withdrawal threshold to stimuli at frequencies of 5 Hz, but not at 250 Hz and 2000 Hz, in wild-type mice (Fig. 1a, 1 h, vehicle: 444 ± 49 μA (n = 9) vs. QX-314 + capsaicin: 1131 ± 65 μA (n = 6), *P* < 0.0001; 3 h, vehicle: 420 ± 52 μA vs. QX-314 + capsaicin: 669 ± 73 μA, *P* = 0.0127). Intraplantar administration of QX-314 (30 mM) alone slightly increased the paw withdrawal threshold to stimuli at frequencies of 5 Hz (1 h, vehicle: 444 ± 49 μA (n = 9) vs. QX-314: 666 ± 75 μA (n = 7), *P* = 0.0245); however, the increase in the paw withdrawal threshold induced by QX-314 alone was much lower than that induced by QX-314 plus capsaicin (Fig. 1a). These results suggested that co-injection of QX-314 and flagellin leads to silencing of Aδ (250 Hz) and Aβ (2000 Hz) fibers, while co-injection of QX-314 and capsaicin silences C fibers (5 Hz) in the wild-type mice.

### Sensory A-fiber silencing in vivo on the mechanical allodynia and the central sensitization in *Tnxb*^−/*−*^ mice

**Figure 2:**
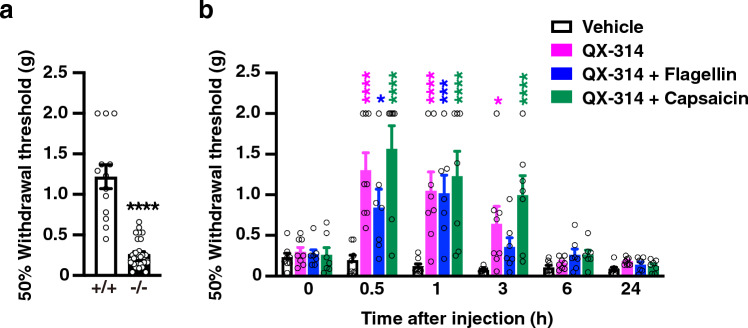
Effect of pharmacological sensory fiber-specific silencing on the mechanical allodynia in *Tnxb*^−/*−*^ mice. (**a**) Mechanical allodynia in *Tnxb*^−/*−*^ mice. 50% paw withdrawal threshold to von Frey filaments was measured in *Tnxb*^+*/*+^ mice (n = 13) and *Tnxb*^−/*−*^ mice (n = 30). ^****^P < 0.0001 versus *Tnxb*^+*/*+^ mice, unpaired *t* test *t*_41_ = 8.952. (**b**) Effects of i.pl. QX-314 (30 mM), QX-314 (30 mM) plus flagellin (0.5 μg), and QX-314 (30 mM) plus capsaicin (10 μg) on the mechanical allodynia in *Tnxb*^−/*−*^ mice. 50% paw withdrawal thresholds to von Frey filaments were assessed in *Tnxb*^−/*−*^ mice by i.pl. administration of vehicle (n = 8), QX-314 (n = 8), QX-314 + flagellin (n = 7), or QX-314 + capsaicin (n = 7). ^****^P < 0.0001, ^***^P < 0.001, ^*^P < 0.05 versus the vehicle-treated value, two-way repeated measurement ANOVA revealed significant time × treatment interaction (F_(15,130)_ = 4.233, *P* < 0.0001) and treatment (F_(3,26)_ = 11.34, *P* < 0.0001) with post hoc Bonferroni’s multiple comparison test. The data are expressed as the mean ± S.E.M.

We examined whether sensory A-fiber silencing in vivo affected the mechanical allodynia in *Tnxb*^−/*−*^ mice. *Tnxb*^−/*−*^ mice significantly decreased in 50% paw withdrawal threshold to von Frey filaments, leading to mechanical allodynia, compared to wild-type mice (Fig. 2a, *Tnxb*^+*/*+^: 1.22 ± 0.15 g (n = 13) vs. *Tnxb*^−/*−*^: 0.26 ± 0.03 g (n = 30), *P* < 0.0001), as with previous our report^9^. Intraplantar co-injection (20 μL) of QX-314 (30 mM) with flagellin (0.5 μg) significantly increased the 50% paw withdrawal threshold at 0.5 h and 1 h after the injection in *Tnxb*^−/*−*^ mice, compared to the vehicle-treated mice (Fig. 2b, 0.5 h, vehicle: 0.19 ± 0.06 g (n = 8) vs. QX-314 + flagellin: 0.84 ± 0.23 g (n = 7), *P* = 0.0108; 1 h, vehicle: 0.12 ± 0.03 g vs. QX-314 + flagellin: 1.02 ± 0.22 g, *P* = 0.0001). This increase in the paw withdrawal threshold induced by QX-314 plus flagellin returned to the pre-administration level at 6 h in *Tnxb*^−/*−*^ mice. These results indicated that A fiber silencing, intraplantar injections of QX-314 plus flagellin, suppressed the mechanical allodynia in *Tnxb*^−/*−*^ mice.

**Figure 3:**
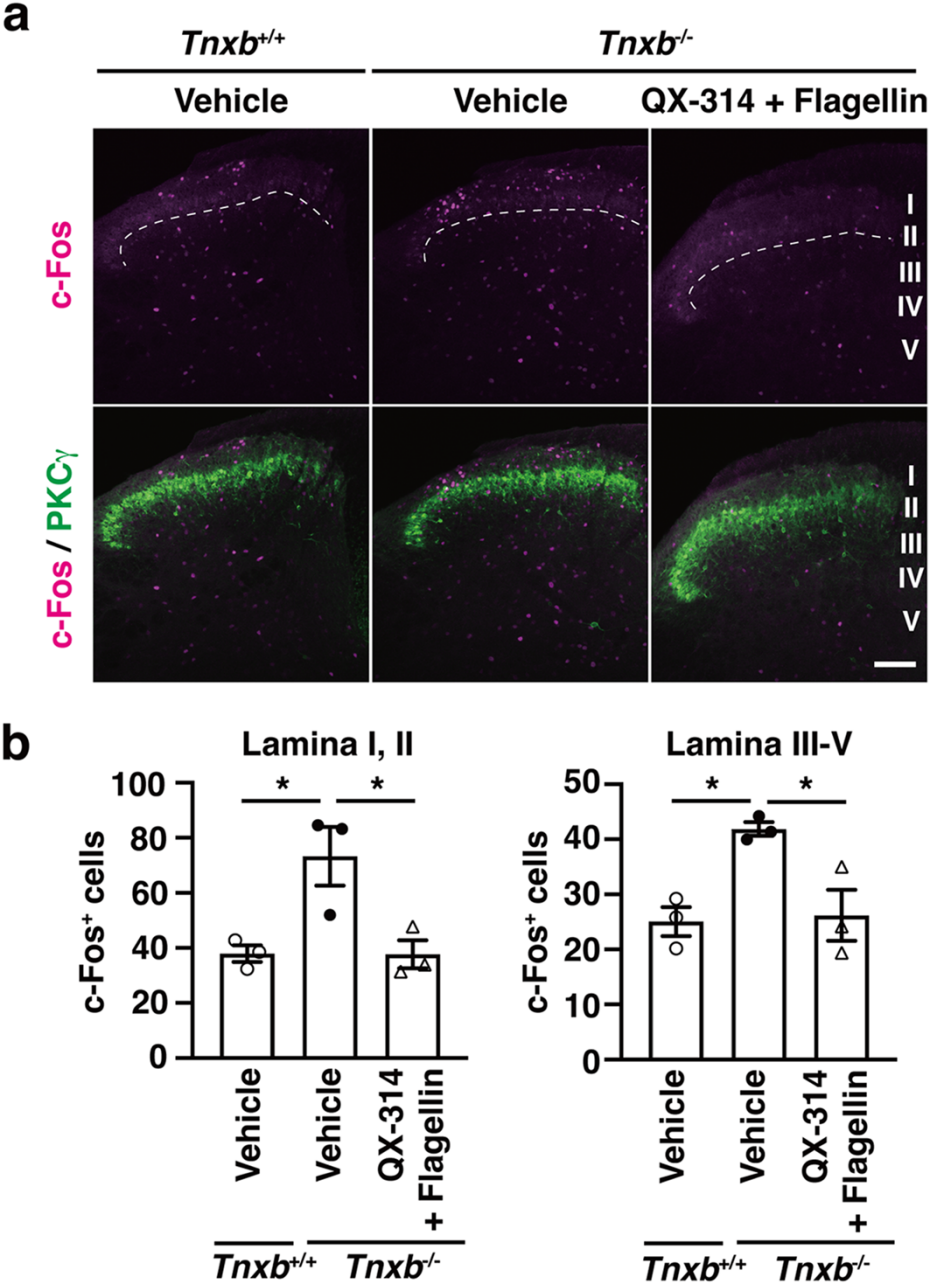
Effect of pharmacological A-fiber silencing on the central sensitization in the spinal dorsal horns of *Tnxb*^−/*−*^ mice. (**a**) Representative fluorescence images of c-Fos (magenta) and PKCγ (green) immunoreactivity in the spinal dorsal horn of *Tnxb*^+*/*+^ and *Tnxb*^−/*−*^ mice injected i.pl. with vehicle or QX-314 (30 mM) + flagellin (0.5 μg). PKCγ was used as a marker of lamina IIi. The dotted lines show the border between lamina II and lamina III. Scale bar, 100 μm. (**b**) The numbers of c-Fos -positive cells in laminae I, II and III–V of the spinal dorsal horn (n = 3 mice, 4–5 sections per mouse). ^*^P < 0.05, one-way ANOVA revealed laminae I, II (F_(2,6)_ = 8.469, *P* = 0.0179) and laminae III-V (F_(2,6)_ = 8.909, *P* = 0.0160) with post hoc Tukey’s multiple comparison test. The data are expressed as the mean ± S.E.M.

Furthermore, we examined the effect of A-fiber silencing on the central sensitization in the spinal dorsal horn of *Tnxb*^−/*−*^ mice. Both the neuronal activation marker c-Fos and the nociceptive-specific activation marker phosphorylated extracellular signal-regulated kinase (p-ERK) in spinal cord are induced by noxious stimuli via C- and Aδ-fibers^14–16^, and by Aβ-fiber activation following nerve injury^17^. c-Fos is also activated by innocuous stimuli via Aβ fiber^14, 18^. In this study, we performed immunostaining of spinal dorsal horn by using c-Fos antibody to clarify the effect of A-fiber silencing. As shown in Fig. 3, the numbers of c-Fos-immunoreactive neurons significantly increased in laminae I and II and in laminae III–V of the spinal dorsal horn in *Tnxb*^−/*−*^ mice injected with vehicle, as compared to *Tnxb*^+*/*+^ mice (laminae I, II, *Tnxb*^+*/*+^: 3 7.9 ± 3.02 (n = 3) vs. *Tnxb*^−/*−*^ with vehicle: 73.3 ± 10.6 (n = 3), *P* = 0.0279; laminae III–V, *Tnxb*^+*/*+^: 25.1 ± 2.62 (n = 3) vs. *Tnxb*^−/*−*^ with vehicle: 41.9 ± 1.24 (n = 3), *P* = 0.0215). When QX-314 (30 mM) plus flagellin (0.5 μg) were injected into the subcutaneous of plantar hind paw in *Tnxb*^−/*−*^ mice, numbers of c-Fos-immunoreactive neurons significantly decreased in laminae I and II and in laminae III–V of ipsilateral spinal dorsal horn at 1 h after the injection, as compared to those of *Tnxb*^−/*−*^ mice injected with vehicle (laminae I, II, *Tnxb*^−/*−*^ with vehicle: 73.3 ± 10.6 (n = 3) vs. *Tnxb*^−/*−*^ with QX-314 + flagellin: 37.7 ± 5.09 (n = 3), *P* = 0.0272 (n = 14), *P* < 0.0001; laminae III–V, *Tnxb*^−/*−*^ with vehicle: 41.9 ± 3.20 (n = 15) vs. *Tnxb*^−/*−*^ with QX-314 + flagellin: 26.2 ± 4.61 (n = 3), *P* = 0.0290). These results indicated that A-fiber silencing, intraplantar injections of QX-314 plus flagellin, blocked the central sensitization in the spinal dorsal horn of *Tnxb*^−/*−*^ mice.

### Sensory C-fiber silencing in vivo on the mechanical allodynia in *Tnxb*^−/*−*^ mice

To characterize the specificity of A-fiber silencing on mechanical allodynia in *Tnxb*^−/*−*^ mice, we determined whether C-fiber silencing by co-injection of QX-314 and capsaicin affects mechanical allodynia in *Tnxb*^−/*−*^ mice. Significant increases in the 50% paw withdrawal threshold were observed following intraplantar injection (20 μL) of QX-314 (30 mM) plus capsaicin (10 μg) or QX-314 (30 mM) alone in *Tnxb*^−/*−*^ mice. Intraplantar injection of QX-314 plus capsaicin significantly increased the paw withdrawal threshold from 0.5 to 3 h after the application in *Tnxb*^−/*−*^ mice compared to vehicle-treated mice (Fig. 2b, 0.5 h, vehicle: 0.19 ± 0.06 g (n = 8) vs. QX-314 + capsaicin (n = 7): 1.56 ± 0.29 g, *P* < 0.0001; 1 h, vehicle: 0.12 ± 0.03 g vs. QX-314 + capsaicin: 1.23 ± 0.31 g, *P* < 0.0001; 3 h, vehicle: 0.08 ± 0.01 g vs. QX-314 + capsaicin: 1.00 ± 0.24 g, *P* < 0.0001). Intraplantar injection of QX-314 alone also increased the paw withdrawal threshold at 0.5 h and 1 h after the application in *Tnxb*^−/*−*^ mice compared to vehicle-treated mice (Fig. 2b, 0.5 h, vehicle: 0.19 ± 0.06 g (n = 8) vs. QX-314 (n = 8): 1.30 ± 0.21 g, *P* < 0.0001; 1 h, vehicle: 0.12 ± 0.03 g vs. QX-314: 1.05 ± 0.23 g, *P* < 0.0001). Taken together, there is no significant difference in the increases of paw withdrawal threshold among QX-314 alone, QX-314 plus flagellin, and QX-314 plus capsaicin (Fig. 2b). These results indicated that QX-314 alone inhibited the mechanical allodynia in *Tnxb*^−/*−*^ mice.

### Effects of sensory fiber silencing in vivo and QX-314 alone on the responses of sensory afferent fibers in *Tnxb*^−/*−*^ mice

**Figure 4:**
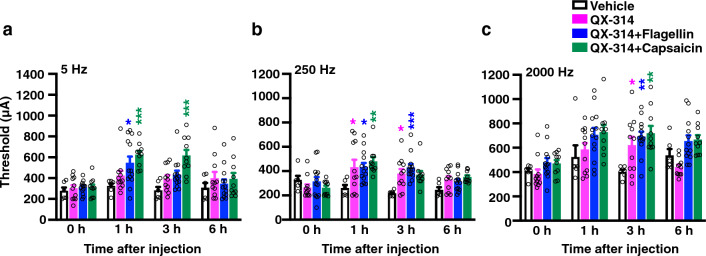
Effects of pharmacological sensory fiber-specific silencing on the responses of sensory afferent fibers using transcutaneous sine wave stimuli in *Tnxb*^−/*−*^ mice. The current threshold represents the minimum intensity (μA) required to produce a paw withdrawal response with sine wave electrical stimulation at 5 Hz (**a**), 250 Hz (**b**), and 2000 Hz (**c**) in mice at the indicated time after i.pl. administration of vehicle, QX-314 (30 mM) plus flagellin (0.5 μg), and QX-314 (30 mM) plus capsaicin (10 μg). Vehicle (n = 6), QX-314 (n = 11), QX-314 + flagellin (n = 13), and QX-314 + capsaicin (n = 10). ^***^P < 0.001, ^**^P < 0.01, ^*^P < 0.05 versus the vehicle-treated value, two-way repeated measurement ANOVA revealed a significant treatment (5 Hz: F_(3,36)_ = 7.007, *P* = 0.0008; 250 Hz: F_(3,36)_ = 4.872, *P* = 0.0060; 2000 Hz: F_(3,36)_ = 6.524, *P* = 0.0012) with post hoc Bonferroni’s multiple comparison test. The data are expressed as the mean ± S.E.M.

We further examined whether sensory fiber silencing in vivo and QX-314 alone affected the responses of sensory afferent fibers by using transcutaneous sine wave stimuli in *Tnxb*^−/*−*^ mice. Similarly to our previous findings^9^, the paw threshold for the 250- and 2000-Hz stimulus responses were significantly reduced in *Tnxb*^−/*−*^ mice compared with those in wild-type mice before the administration of drugs (Table 1, 250 Hz, wild-type: 401 ± 17 μA (n = 31) vs. *Tnxb*^−/*−*^: 288 ± 16 μA (n = 40), *P* < 0.0001; 2000 Hz, wild-type: 511 ± 23 μA vs. *Tnxb*^−/*−*^: 438 ± 19 μA, *P* = 0.0164). As shown in Fig. 4b,c, intraplantar administration of QX-314 plus flagellin significantly increased the threshold at frequencies of 250 Hz and 2000 Hz in *Tnxb*^−/*−*^ mice, compared to vehicle administration (250 Hz: 1 h, vehicle: 259 ± 28 μA (n = 6) vs. QX-314 + flagellin: 432 ± 36 μA (n = 13), *P* = 0.0119; 3 h, vehicle: 216 ± 9.7 μA vs. QX-314 + flagellin: 429 ± 30 μA, *P* = 0.0009; 2000 Hz: 3 h, vehicle: 400 ± 20 μA vs. QX-314 + flagellin: 690 ± 42 μA, *P* = 0.0024). Intraplantar administration of QX-314 plus capsaicin also increased the paw withdrawal threshold in the stimuli at frequencies of 250 Hz and 2000 Hz in *Tnxb*^−/*−*^ mice (250 Hz: 1 h, vehicle: 259 ± 28 μA (n = 6) vs. QX-314 + capsaicn: 478 ± 36 μA (n = 10), *P* = 0.0012; 2000 Hz: 3 h, vehicle: 400 ± 20 μA vs. QX-314 + capsaicn: 717 ± 63 μA, *P* = 0.0013), different from that of wild-type mice (Fig. 1b,c). Furthermore, the threshold level induced by the co-application of QX-314 with flagellin or capsaicin was similar to that induced by QX-314 alone in the stimuli at frequencies of 250 Hz and 2000 Hz in *Tnxb*^−/*−*^ mice. The QX-314 alone-induced threshold increase was approximately 1.6–1.8-fold higher than the vehicle-induced one in 1 h and 3 h after the administration at frequencies of 250 Hz (1 h, vehicle: 259 ± 28 μA (n = 6) vs. QX-314: 430 ± 64 μA (n = 11), *P* = 0.0172, 3 h, vehicle: 216 ± 9.8 μA vs. QX-314: 378 ± 40 μA, *P* = 0.0272) and in 3-h at frequencies of 2000 Hz (vehicle: 400 ± 20 μA vs. QX-314: 620 ± 69 μA, *P* = 0.0497) in *Tnxb*^−/*−*^ mice. Consistent with the results of paw withdrawal threshold, QX-314 alone, QX-314 plus flagellin, and QX-314 plus capsaicin affected the thresholds for A-fibers responded by the 250- and 2000-Hz stimulus in *Tnxb*^−/*−*^ mice.

**Table 1:** Paw withdrawal thresholds in responses to sine-wave electrical stimuli in *Tnxb*^+/+^ and *Tnxb*^−/*−*^ mice before the administration of drugs.

		Threshold (μA)		
		5 Hz	250 Hz	2000 Hz
*Tnxb*^+/+^	(n = 31)	337 ± 15	401 ± 17	511 ± 23
*Tnxb*^−/*−*^	(n = 40)	302 ± 13	288 ± 16****	438 ± 19*

The current threshold represents the minimum intensity (μA) required to produce a paw withdrawal response with sine wave electrical stimulation at 5, 250, and 2000 Hz. The data ****P < 0.0001, *P < 0.05 versus the *Tnxb*^+/+^ value, unpaired *t* test 5 Hz: *t*_69_ = 1.744, 250 Hz: *t*_69_ = 4.672, 2000 Hz: *t*_69_ = 2.460. The data are expressed as the mean ± S.E.M. from experiments using *Tnxb*^+/+^ (Fig. 1) and *Tnxb*^−/*−*^ mice (Fig. 4).

In the response to transcutaneous sine wave stimuli at a frequency of 5 Hz, there was no significant difference between wild-type and *Tnxb*^−/*−*^ mice before the administration of drugs (Table 1, wild-type: 337 ± 15 μA (n = 31) vs. *Tnxb*^−/*−*^: 302 ± 13 μA (n = 40), *P* = 0.0857). Intraplantar administration of QX-314 plus capsaicin significantly increased at 1 h and 3 h compared to vehicle administration in *Tnxb*^−/*−*^ mice (Fig. 4a, 1h, vehicle: 323 ± 26 μA (n = 6) vs. QX-314 + capsaicin: 624 ± 36 μA (n = 10), *P* = 0.0007, 3 h, vehicle: 280 ± 35 μA vs. QX-314 + capsaicin: 615 ± 56 μA, *P* = 0.0001); however, the maximum increase observed at 1 h in *Tnxb*^−/*−*^ mice was substantially lower than that in wild-type mice (Fig. 1a). This result suggests that the TNX deficiency affects the C-fiber silencing mechanisms induced by QX-314 plus capsaicin. Furthermore, intraplantar administration of QX-314 plus flagellin, but not QX-314 alone, increased the threshold at frequencies of 5 Hz, in addition to frequencies of 250 Hz and 2000 Hz, in *Tnxb*^−/*−*^ mice (Fig. 4a, 1 h, vehicle: 323 ± 26 μA (n = 6) vs. QX-314 + flagellin: 544 ± 62 μA (n = 13), *P* = 0.0160), indicating that administration of QX-314 plus flagellin also affects the C-fiber response in *Tnxb*^−/*−*^ mice.

### QX-314-induced inhibition of mechanical allodynia though TLR5-dependent activation in *Tnxb*^−/*−*^ mice

**Figure 5:**
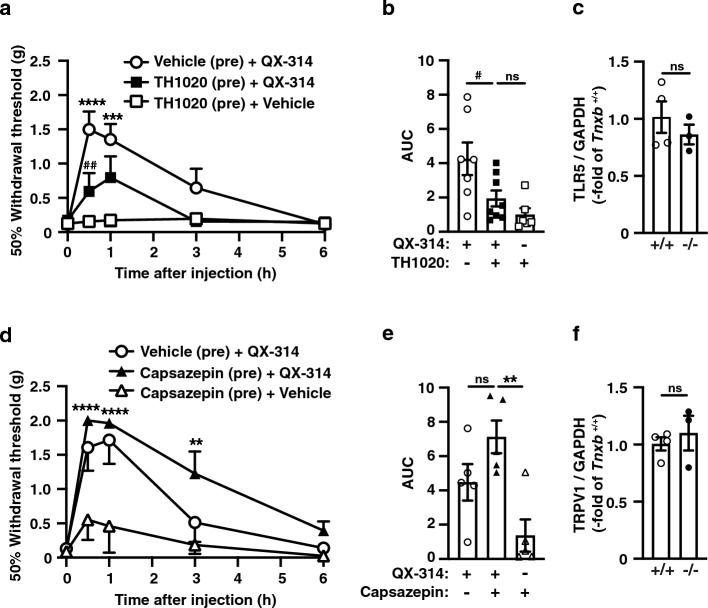
Blocking of the QX-314-induced inhibition of mechanical allodynia by a TLR5/flagellin complex antagonist TH1020 in *Tnxb*^−/*−*^ mice. (**a**) TH1020 (10 nmol, 4.5 μg) or vehicle was intraplantar administrated at 30-min before QX-314 (60 mM; 10 μL) injection in *Tnxb*^−/*−*^ mice. 50% paw withdrawal thresholds to von Frey filaments were assessed in *Tnxb*^−/*−*^ mice by i.pl. administration of TH1020 (n = 6), vehicle(pre) + QX-314 (n = 7), or TH1020 + QX-314 (n = 8). ^##^*P* < 0.01 versus the vehicle(pre) + QX-314 treated value, ^****^P < 0.0001, ^***^P < 0.001 versus the TH1020(pre) + vehicle treated value, two-way repeated measurement ANOVA revealed significant time × treatment interaction (F_(8,72)_ = 4.712, *P* = 0.0001) and treatment (F_(2,18)_ = 7.321, *P* = 0.0047) with post hoc Bonferroni’s multiple comparison test. (**b**) AUC based on data from (**a**) were represented. ^**^P < 0.01, ^*^P < 0.05, one-way ANOVA (F_(2,18)_ = 6.257, *P* = 0.0086) with post hoc Tukey’s multiple comparison test. (**c**) Expression of TLR5 mRNA in dorsal root ganglion of wild-type and *Tnxb*^−/*−*^ mice. (**d**) A TRPV1 antagonist capsazepine (27 nmol, 10 μg) or vehicle was intraplantar administrated at 30-min before QX-314 (60 mM; 10 μL) injection in *Tnxb*^−/*−*^ mice. 50% paw withdrawal thresholds to von Frey filaments were assessed in *Tnxb*^−/*−*^ mice by i.pl. administration of capsazepine(pre) + vehicle (n = 5), vehicle(pre) + QX-314 (n = 5), or capsazepine(pre) + QX-314 (n = 5). ^****^P < 0.0001, ^***^P < 0.001, ^**^P < 0.01 versus the capsazepine(pre) + vehicle treated value, two-way repeated measurement ANOVA revealed significant time × treatment interaction (F_(8,48)_ = 4.156, *P* = 0.0008) and treatment (F_(2,12)_ = 9.906, *P* = 0.0029) with post hoc Bonferroni’s multiple comparison test. (**e**) AUC based on data from (**d**) were represented. ^**^P < 0.01, ^*^P < 0.05, one-way ANOVA (F_(2,12)_ = 8.535, *P* = 0.0049) with post hoc Tukey’s multiple comparison test. (**f**) Expression of TRPV1 mRNA in dorsal root ganglion of wild-type and *Tnxb*^−/*−*^ mice. The data are expressed as the mean ± S.E.M.

Intraplantar co-injection of QX-314 with flagellin, but not QX-314 alone, increased the paw withdrawal thresholds of Aβ- and Aδ-fibers in wild-type mice (Fig. 1b,c). On the other hand, intraplantar injection of QX-314 alone led to the increase of thresholds responded for Aβ-and Aδ-fibers (Fig. 4b,c) and inhibition of mechanical allodynia (Fig. 2b) in *Tnxb*^−/*−*^ mice. Therefore, we hypothesized that the effects of QX-314 alone are mediated by constitutive activation of TLR5 in *Tnxb*^−/*−*^ mice. To investigate this possibility, we examined the effect of a potent and selective TLR5/flagellin complex antagonist, TH1020, on mechanical allodynia in *Tnxb*^−/*−*^ mice. Pre-administration of TH1020 markedly suppressed the QX-314-induced increase of 50% paw withdrawal threshold (Fig. 5a), and the significant decrease induced by TH1020 was observed at 0.5 h after the injection of QX-314 (vehicle pre-injection + QX-314: 1.50 ± 0.26 g (n = 7) vs. TH1020 pre-injection + QX-314: 0.59 ± 0.26 g (n = 8), *P* = 0.0033). Intraplantar injection of TH1020 (10 nmol, 4.5 μg) did not affect the TNX-deficiency-induced mechanical allodynia. Area under the curve (AUC) of 50% paw withdrawal threshold induced by QX-314 alone was also inhibited by pre-administration of TH1020 in *Tnxb*^−/*−*^ mice (Fig. 5b). In contrast to TH1020, pre-administration of 10 nmol (4.5 μg) capsazepine, a TRPV1 antagonist, had no significant effect on QX-314-induced inhibition of mechanical allodynia in *Tnxb*^−/*−*^ mice (Fig. 5d,e). We analyzed the expression of TLR5 and TRPV1 mRNAs in dorsal root ganglion, which contained soma of sensory neurons, of *Tnxb*^−/*−*^ mice. There is no significant difference in mRNA expression of TRLR5 and TRPV1 between wild-type and *Tnxb*^−/*−*^ mice (Fig. 5c and f). These results indicated that inhibition of mechanical allodynia with QX-314 alone is mediated by constitutive activation of TLR5 in *Tnxb*^−/*−*^ mice.

## Discussion

In the present study, we revealed that the mechanical allodynia and central sensitization of the spinal dorsal horn were medicated by the hypersensitization of Aβ and Aδ fibers in *Tnxb*^−/*−*^ mice, by using pharmacological sensory fiber silencing. A-fiber silencing, intraplantar (i.pl.) co-administration of QX-314 and flagellin, significantly increased the paw withdrawal threshold of Aβ and Aδ fibers to transcutaneous sine wave stimuli at frequencies of 250 Hz and 2000 Hz, respectively, in wild-type mice. Intraplantar co-administration of QX-314 and flagellin significantly increased the paw withdrawal threshold of Aβ and Aδ fibers in *Tnxb*^−/*−*^ mice, and it inhibited the mechanical allodynia and spinal neuronal activation in *Tnxb*^−/*−*^ mice. Notably, in *Tnxb*^−/*−*^ mice, but not wild-type mice, i.pl. administration of QX-314 alone significantly increased the paw withdrawal threshold of Aβ and Aδ fibers and subsequent mechanical allodynia. Thus, the activation of Aβ and Aδ fibers led to the TNX deficiency induced-mechanical allodynia. Furthermore, the QX-314-induced inhibition of mechanical allodynia was reduced by TLR5 antagonist TH1020, suggesting that it is mediated via constitutive activation of TLR5.

Blocking A-fibers can be specifically achieved by using TLR5 ligand flagellin and the membrane-impermeable sodium channel blocker QX-314^10^. Likewise, i.pl. co-administration of QX-314 and flagellin significantly increased the paw withdrawal threshold of Aβ and Aδ fibers to transcutaneous sine wave stimuli at frequencies of 250 Hz and 2000 Hz in wild-type mice (Fig. 1). The QX-314 plus flagellin-induced increase of threshold to stimuli at frequencies of 250 Hz and 2000 Hz was also observed in *Tnxb*^−/*−*^ mice (Fig. 4). We have previously reported that TNX deficiency induces mechanical allodynia and central sensitization in the dorsal horn of the spinal cord, in addition to hypersensitization of myelinated Aβ and Aδ fibers^9^. A-fiber silencing, i.pl. co-administration of QX-314 and flagellin, significantly inhibited the mechanical allodynia (Fig. 2) and the increase of c-Fos positive neurons of spinal dorsal horn (Fig. 3). Surprisingly, intraplantar administration of QX-314 alone significantly increased the paw withdrawal threshold to stimuli at frequencies of 250 Hz and 2000 Hz in *Tnxb*^−/*−*^ mice (Fig. 4) but not wild-type mice (Fig. 1). Intraplantar administration of QX-314 alone inhibited the mechanical allodynia in *Tnxb*^−/*−*^ mice (Fig. 2). These results suggest that hypersensitization of myelinated Aβ and Aδ fibers responded to stimuli at frequencies of 250 Hz and 2000 Hz led to the mechanical allodynia.

QX-314 is a membrane-impermeable voltage-gated sodium channels blocker that blocks these channels by binding to their intracellular domains. Although the extracellular application of QX-314 theoretically has no effect on the activity of sodium channels, QX-314 has been shown to enter the cytoplasm of cells by activating the TRPV1 channel^11^. Intraplantar co-administration of QX-314 (2% (approximately 60 mM), 10 μL) and capsaicin (1 μg/μL, 10 μL) increased the mechanical threshold for paw withdrawal using von Frey filaments, whereas i.pl. administration of QX-314 alone had no significant effect on the mechanical threshold. In this study, i.pl. administration of QX-314 (30 mM) without a TRPV1 agonist inhibited mechanical allodynia in *Tnxb*^−/*−*^ mice (Fig. 2b). Consistent with this result, QX-314 (2%, 60 mM) alone applied i.pl. to UV-burn-induced inflamed paws has an inhibitory effect on paw mechanical sensitivity^19^. During tissue damage and inflammation, inflammatory mediators such as ATP, prostaglandins, and bradykinin released from surrounding damaged or inflamed tissues and the acidic environment surrounding the inflamed tissues have been reported to sensitize the response of TRPV1^20, 21^. Furthermore, systemic intraperitoneal administration of QX-314 (1–3 mg/kg) alone reduced bone cancer pain-related behaviors by inhibiting TRPV1-expressing afferents^22^. In a bone cancer pain model, tumor growth induces inflammation and an acidic environment is induced by activated osteoclasts^23^. These reports suggest that the inhibitory effects of QX-314 alone are mediated by TRPV1 activation. However, the TRPV1 antagonist capsazepine failed to suppress the inhibition of mechanical allodynia induced by i.pl. administration of QX-314 alone in *Tnxb*^−/*−*^ mice (Figs. 5d,e), suggesting that the inhibitory mechanisms of QX-314 alone are independent of TRPV1 channel activation. We identified a novel mechanism of action for i.pl. QX-314 alone, in which TLR5 was involved in inhibiting mechanical allodynia in *Tnxb*^−/*−*^ mice. A potent and selective TLR5/flagellin complex antagonist, TH1020, blocked the QX-314-induced inhibition of mechanical allodynia in *Tnxb*^−/*−*^ mice (Figs. 5a,b). Flagellin, a TLR5 agonist, induces the entry of extracellular QX-314 into the cytoplasm via unidentified pores such as channel^10^. Thus, TNX-deficiency may lead to constitutive activation of TLR5 and subsequent cellular signaling.

Axonal polyneuropathy occurs in TNX-deficient EDS patients^3, 5, 24^. Nerve conduction studies were abnormal in 80% of TNX-deficient EDS patients, and fulfilled the criteria of sensorimotor axonal polyneuropathy in 40% of the patients^5^. Electromyography shows TNX-deficient EDS patients had a predominant neurogenic and a mixed myogenic-neurogenic patterns^5^. Neuropathic symptoms including paresthesia are frequently observed in patients with TNX-deficient EDS^3, 5^. Voermans et al*.* reported histological changes in sciatic nerves such as mildly smaller inner and outer diameters of the myelinated fiber and reduced collagen fibril density of the endoneurium in *Tnxb*^−/*−*^ mice^25^. We have reported that TNX deficiency induces hypersensitivity of myelinated Aβ and Aδ fibers, whereas it has no effect on C-fiber response under the basal condition^9^. In this study, A-fiber silencing, co-administration of QX-314 and flagellin, affected the paw withdrawal threshold of C fiber to stimuli at frequencies of 5 Hz in *Tnxb*^−/*−*^ mice (Fig. 4a). This finding raises a possibility that A fibers partially cross over C fiber in sensory fibers of *Tnxb*^−/*−*^ mice. Fiber interaction such as ephaptic and cross-excitation is thought to be one of peripheral neuropathy mechanisms whereby nociceptive fibers such as C- and Aδ-fibers could be stimulated by activity in low-threshold mechanoreceptors in non-nociceptive Aβ fibers^26–29^. TNX-deficiency may induce physical crosstalk between A fibers and C fibers. In addition, C-fiber silencing effect of the paw withdrawal threshold for responses to 5 Hz stimuli in *Tnxb*^−/*−*^ mice was lower than that in wild-type mice, although administration of QX-314 plus capsaicin significantly increased the threshold at a frequency of 5 Hz in both wild-type and *Tnxb*^−/*−*^ mice (Figs. 1a and 4a). There was no difference in the threshold for responses to 5 Hz stimuli at pre-administration between wild-type and *Tnxb*^−/*−*^ mice. These results suggest that TNX deficiency affects the C-fiber silencing mechanisms by using QX-314 and capsaicin. The expression level of the capsaicin receptor TRPV1 mRNA in dorsal root ganglion of *Tnxb*^−/*−*^ mice was similar to that of wild-type mice (Fig. 5f). TNX-deficient mice showed normal behavioral response to the noxious thermal stimulation in hot plate-test^9^, whereas TRPV1-deficient mice showed impaired behavioral response to the noxious thermal stimulation compared to wild-type mice^30^. Taken together, TNX deficiency inhibits the QX-314 plus capsaicin-evoked C-fiber silencing through an undetermined mechanism independent of TRPV1. The pathophysiological mechanism of peripheral neuropathy in patients with TNX-deficient EDS seems to be related to the dysfunction of unmyelinated C-fibers, in addition to the hypersensitivity of myelinated Aβ and Aδ fibers.

In conclusions, we demonstrated that the TNX deficiency-induced mechanical allodynia is mediated by A-fiber hypersensitivity using pharmacological sensory fiber silencing with co-administration of QX-314 and flagellin. In addition, QX-314 alone led to the silencing of A-fiber responses and inhibition of mechanical allodynia through the constitutive activation of TLR5 in *Tnxb*^−/*−*^ mice. Our findings will be useful for managing neurological complications in patients with EDS.

## Methods

### Animals

All experiments were approved by the Animal Experimentation Committee of the Osaka Institute of Technology and were performed in accordance with the National Institutes of Health Guide for the Care and Use of Laboratory Animals and the ethical guidelines of the Ethics Committee of the International Association for the Study of Pain. This study was conducted in accordance with ARRIVE guidelines. *Tnxb*^−/*−*^ mice were generated as previously reported^31^, and they were backcrossed with C57BL/6 J mice for ten generations. Age-matched C57BL/6 J mice were used as wild-type (*Tnxb*^+*/*+^) mice. Seven to 9-week-old male mice were used for the experiments, because of the marked sex-related differences in mechanisms of pain hypersensitivity^32^. Animals were housed under conditions of a 12-h light/12-h dark cycle and at a constant temperature of 22 ± 2 °C. Animals were allowed free access to food and water prior to testing.

### Administration of drug and antibody

Capsaicin and capsazepin were purchased from Fujifilm Wako Pure Chemical (Osaka, Japan), flagellin from Sigma-Aldrich (St. Louis, MO, USA), QX-314 from Abcam (Cambridge, UK), and TH1020 from MedChemExpress (Monmouth Junction, NJ, USA). Capsaicin (1 mg/mL) was dissolved in saline containing 20% ethanol and 5% Tween 20. Flagellin (0.1 mg/mL) was dissolved in distilled water, TH1020 (8.8 mM, 4 mg/mL) was dissolved in dimethyl sulfoxide and diluted to a final concentration with saline. QX-314 was dissolved in a saline solution. Capsazepine (1 mg/mL) was dissolved in saline containing 5% dimethyl sulfoxide and 5% Tween 20. Mice were injected subcutaneously into the plantar surface of the hind paw with 20 μL of QX-314 (30 mM), QX-314 (30 mM) plus flagellin (0.5 μg in 20 μL), QX-314 (30 mM) plus capsaicin (10 μg in 20 μL), and TH1020 (10 nmol, 4.5 μg), using 27-gauge stainless steel needle attached to a microsyringe. For pre-administration of TH1020 or capsazepine, mice were subcutaneously injected with 10 μL of TH1020 (10 nmol in 10 μL), capsazepine (10 μg in 10 μL), or vehicle, and then 10 μL of QX-314 (60 mM) was administrated 30 min after administration. All drug administration experiments were performed by investigators blinded to the drugs.

### von Frey test

Mice were randomly placed in glass chambers on a mesh floor. Mice were habituated to the test environment for 30 min. Mechanical nociception was assessed using the up-down method with von Frey filaments^33^. Mechanical sensitivity was evaluated using calibrated von Frey filaments (0.02–2.0 g). The first stimulus was always a 0.4-g filament. When a paw withdrawal reflex of the paw was elicited, the next lower-rated filament was applied, and when there was no response, the next higher-rated filament was used. After the first change in the response direction, four additional measurements were carried out, and the 50% withdrawal threshold was calculated using the up-down method^33^.

### Electrical stimulation-induced paw withdrawal test

The electrical stimulation-induced paw withdrawal test was performed as previously described^22^. Briefly, electrodes (3 mm in diameter) were attached to the left plantar and dorsal surfaces of the hind paws of each mouse. Transcutaneous nerve stimuli using each of three sine wave currents (5, 250, and 2000 Hz) were applied for 3 s through the electrodes. The current intensity increased gradually, and the minimum current intensity of the paw withdrawal response was defined as the paw withdrawal threshold. Transcutaneous nerve stimuli using each sine wave currents were applied to the paw at 5-min intervals.

### Immunohistochemistry

Immunohistochemistry was performed as previously described^34^. Briefly, mice were deeply anesthetized using sodium pentobarbital and then intracardially perfused with phosphate-buffered saline (PBS) followed by 4% paraformaldehyde in 0.1 M phosphate buffer (pH 7.4). The lumbar spinal cord was dissected, fixed in 4% paraformaldehyde overnight, and cryoprotected in 30% sucrose overnight. Spinal cord sections (40 μm thick) were prepared using a sliding microtome. The free-floating spinal cord was blocked with PBS containing 10% normal goat serum and 0.2% Triton X-100 and then incubated with primary antibodies overnight. The primary antibodies included rabbit anti-c-Fos (1:1000, Cell Signaling Technology, Danvers, MA, USA #2250), and guinea pig anti-protein kinase Cγ (PKCγ, 1:250, Frontier Institute, Hokkaido, Japan, #AB2571826). The immune complexes were visualized using Alexa 546-labeled anti-rabbit IgG (1:1000, ThermoFisher Scientific, Waltham, MA, USA #A11035), and Alexa 488-labeled anti-guinea pig IgG (1:800, Jackson ImmunoResearch Laboratories, West Grove, PA, USA, #706-545-148) secondary antibodies. Digital images were captured using a Nikon A1 laser-scanning confocal microscope equipped with an argon HeNe1 laser and appropriate filter (Nikon Corporation, Tokyo, Japan). Sections of the spinal cord were concurrently immunostained, and images were captured under the same conditions. c-Fos-positive cells were counted using the NIH ImageJ software. Background intensity (arbitrary units) was measured in the white matter area of the spinal cord. A cell was counted as positive cells if its intensity level was at least twice that of the background intensity level and if a single cell body was clearly defined. c-Fos immunoreactivity was quantified in 4–5 sections from each mouse (n = 3 mice). The numbers of positive cells from each mouse were expressed as the mean ± S.E.M of the number in the indicated area (laminae I, II, and III–V) of the half spinal dorsal horn image.

## Quantitative real-time PCR

Total RNAs were extracted from mouse tissues using TRIzol reagent (ThermoFisher Scientific), and the first-strand cDNA was synthesized from 0.8 μg of total RNA by using a ReverTra Ace (Toyobo, Osaka, Japan). First-strand cDNA was amplified in a GoTaq qPCR master mix (Promega, Madison, WI, USA) with specific primers, and the amplified products were detected with a QuantStudio 1 real-time PCR system (Applied Biosystem, Waltham, MA, USA). The sequences of primers were as follows: 5′-TTCCCGCCTCCAGATTCTTT-3′ and 5′-CAAAGCAGGGTCAGGAGAGA-3′ for TLR5; 5′-TCATTGCTCTCATGGGCGAGACTG-3′ and 5′-TATGCCTATCTCGAGTGCTTGCGT-3′ for TRPV1; 5′-AACTTTGGCATTGTGGAAGG-3′ and 5′-ACACATTGGGGGTAGGAACA-3′ for glyceraldehyde-3-phosphate dehydrogenase (GAPDH)^35^. The real-time PCR-amplifications were performed as follows: 1 cycle at 95 °C for 10 min followed by 40 cycles at 95 °C for 15 s and 60 °C for 1 min. The relative mRNA levels were calculated from the threshold cycle (Ct) values according to the ΔΔCt method. The Ct values were normalized to those of GAPDH, as an internal control.

### Statistical analysis

Statistical analyses were performed using Prism 8 (GraphPad Software Inc., La Jolla, CA, USA). Statistical analysis was performed using the unpaired Student’s *t*-test (two-tailed) for comparisons between the two groups. Group means were compared using a one-way ANOVA with a post hoc Tukey’s multiple comparison test and two-way repeated measures ANOVA with a post hoc Bonferroni test. Differences were considered statistically significant at *P* < 0.05. Data are expressed as the mean ± S.E.M.

## Data Availability

The datasets generated in this study are available from the corresponding author on reasonable request.
